# The complete mitochondrial genome of *Aorianigripes* (Coleoptera, Eumolpidae, Eumolpinae) and its phylogenetic status

**DOI:** 10.3897/BDJ.10.e93591

**Published:** 2022-12-19

**Authors:** Chenju Yang, Chunyan Jiang, Shaoxiong Wu, Xiayu Feng, Zhengwen Yu

**Affiliations:** 1 School of Life Sciences, Guizhou Normal University, Guiyang, China School of Life Sciences, Guizhou Normal University Guiyang China

**Keywords:** *Aorianigripes*, *Ampelopsis*, mitochondrial genome, phylogenetic analysis

## Abstract

*Aorianigripes* (Baly, 1860) is one of the main pests of grapes, mainly damaging leaves, petioles and shoots and seriously affecting plant growth and development. Recently, this pest was found to damage the leaves of *Ampelopsisgrossedentata*, *Ampelopsismegalophylla*, *Ampelopsischaffanjonii* and *Ampelopsiscantoniensis.* However, the phylogenetic relationships of *A.nigripes* and other related family members are unclear. In this study, we sequenced and analysed the complete mitogenome of *A.nigripes* for the first time. The mitogenome of *A.nigripes* is circular and 17,306 bp in size, consisting of 13 protein-coding genes (PCGs), 22 transfer RNA genes (tRNAs) and two ribosomal RNA genes (rRNAs). The base composition of the *A.nigripes* mitogenome is 41.70% A, 33.76% T, 9.01% G and 15.53% C. The phylogenetic analysis showed that *A.nigripes* was clustered together with *Basileptafulvipes* and *Colasposomadauricum*.

## Introduction

**Figure 1.**
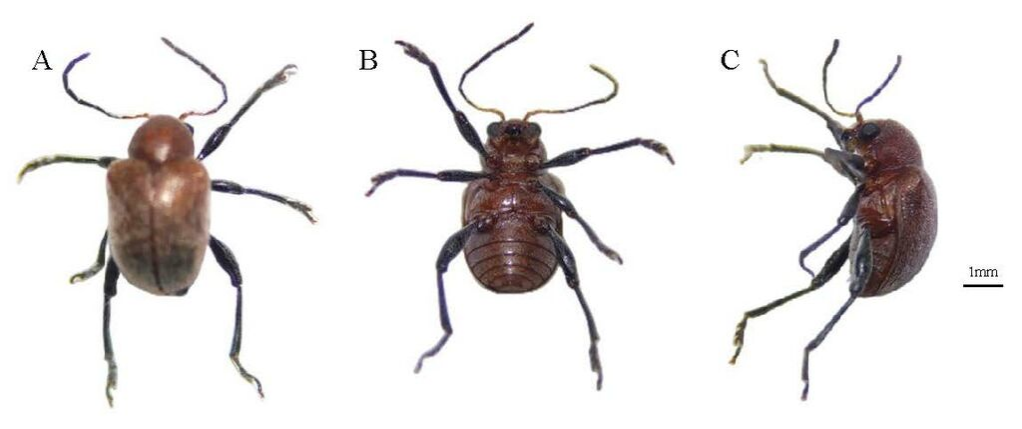
*Aorianigripes* (Baly, 1860), femal. **A** dorsal view; **B** ventral view; **C** lateral view. Scale bar: 1 mm.

**Figure 2.**
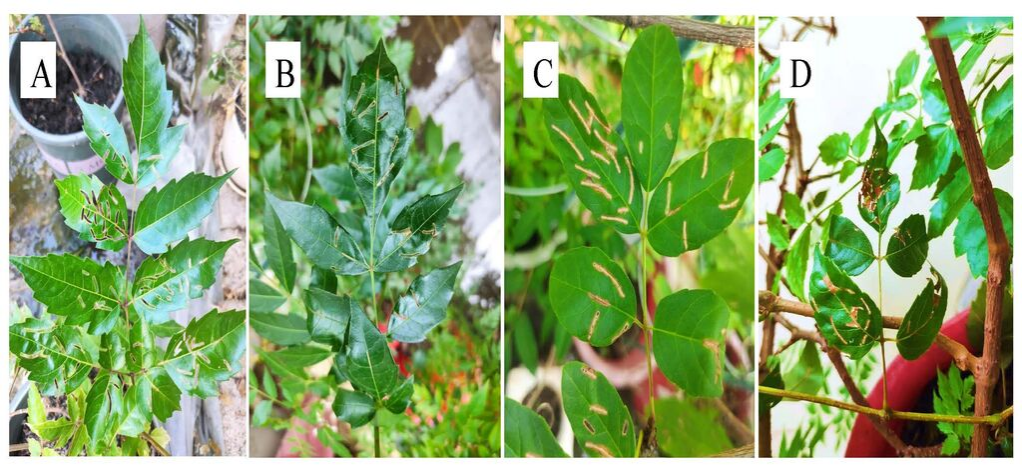
Plants of the genus *Ampelopsis* were damaged by *A.nigripes. Ampelopsisgrossedentata* **(A)**; *Ampelopsismegalophylla* **(B)**; *Ampelopsischaffanjonii* **(C)**; *Ampelopsiscantoniensis* **(D)**.

*Aorianigripes* (Baly, 1860) belongs to the Coleoptera, Eumolpidae, widely distributed in China, Vietnam, Laos, Cambodia, Myanmar, Thailand, India, Indonesia and other regions (Tan et al. 2016). *A.nigripes* with a brownish-yellow, brownish-red to chestnut-brown body colour, head and thorax with black, antennae black, leg black with slightly dyed red, body length 4.5 ~ 7.2 mm and body width 2.8 ~ 4.2 mm (Fig. 1), has been reported to cause serious damage to the family Vitaceae and is commonly found on grapes and wild grapes, *Parthenocisushimalayana* (Qin et al. 2004), Crystal Grape (Zhou et al. 2015) and *Quercus* (Hua 2002), mainly as an adult on leaves, petioles and shoots, feeding on the adaxial leaf flesh of leaves and the epidermis of branches and petioles, causing transparent feeding strips on the leaves (Wang et al. 2011, Zhou et al. 2015). Wang et al. (2011) used 3% Acetamiprid，4.5% Beta cypermethrin and 2.5% Bifenthrin to control it. Recently, it was found to damage the leaves of *Ampelopsisgrossedentata*, *Ampelopsismegalophylla*, *Ampelopsischaffanjonii* and *Ampelopsiscantoniensis* (Fig. 2), which are rich in flavonoids and have clearing heat and detoxifying, antioxidant and antitumour properties (Kou and Chen 2012, Gao et al. 2014, Xie et al. 2014, Tong et al. 2015, Xie et al. 2015, Ye et al. 2015, Jin et al. 2016, Chu 2018, Huang et al. 2018, Thu et al. 2020). There has been very little research to date on the occurrence and control techniques of *A.nigripes* on medicinal and economic plants of the genus *Ampelopsis.* It is worthwhile to extract volatile secondary metabolites from host plants, screen attractive "surrogate" plants and transfer affected hosts for green control.

Regarding the taxonomic status of the family Eumolpidae, Chen (Chen 1940a, Chen 1940b, Chen 1940c, Chen 1964) systematically studied the leaf beetles of China. It was proposed that the five subfamilies (Eumolpinae, Lamprosomatinae, Chlamisinae, Clytrinae and Cryptocephalinae) should form the family Eumolpidae. Tan et al. (2016) agreed with Chen's view that their adult morphologic characteristics and larval life habits were obviously related and they should form an independent family. Previous studies have analyzed the phylogeny and evolution of the superfamily Chrysomeloidea, but the genetic and evolutionary relationships among the species of Eumolpidae remain unclear (Haddad and Mckenna 2016, Nie et al. 2020, Nie et al. 2021). Here, we report the complete mitogenome sequence of *A.nigripes* and its phylogenetic position within the superfamily Chrysomeloidea which will provide a valuable resource for the better study of *A.nigripes* in the future.

## Materials and methods

The samples of *A.nigripes* were collected by Chenju Yang with hand-picking from *A.grossedentata* Germplasm Resource Nursery of China, which is a medicinal plant nursery in Guizhou Normal University, Guiyang City, Guizhou Province, China (26°38'48"N, 106°63'26"E). The specimen was deposited at the School of Life Sciences, Guizhou Normal University (Chenju Yang; e-mail yangchenju1123@163.com) under voucher number GZNUYCJ202201001. Total genetic DNA was extracted using the CTAB method (cetyltrimethyl ammonium bromide). The whole-genome shotgun (WGS) strategy was adopted to construct a library (size 350 bp), then Illumina NovaSeq sequencing platform was used for double-ended sequencing according to the manufacturer’s protocol. The raw data obtained were filtered using SOAPnuke v.1.3.0 to obtain 4.2 GB clean reads and saved in fastq format (Ewing and Green 1998, Cock et al. 2010, Jiang et al. 2011). Afterwards, the filtered reads were assembled using the programme SPAdes v.3.14.0 (Bankevich et al. 2012) with *Colasposomadauricum* (GenBank accession number: NC_057218) as the initial reference genome and the assembled mitogenome was annotated using the online software MITOS2 (http://mitos2.bioinf.uni-leipzig.de/index.py) (Bernt et al. 2013). Finally, the complete mitogenome was submitted to GenBank with accession number NC_065028.

Based on the 13 PCGs, phylogenetic relationships were analysed for 56 species from the superfamily Chrysomeloidea, with *Anoplophoraglabripennis* (NC_008221) and *Batoceralineolata* (NC_022671) as outgroups, which were downloaded from GenBank to construct phylogenetic trees with *A.nigripes*, based on the Maximum Likelihood (ML) method. The ML tree, based on the GTR + G + I model, was constructed using IQ-tree v.2.04 with 1000 ultrafast bootstrap replicates (Nguyen et al. 2015).

## Results and Discussion

**Figure 3.**
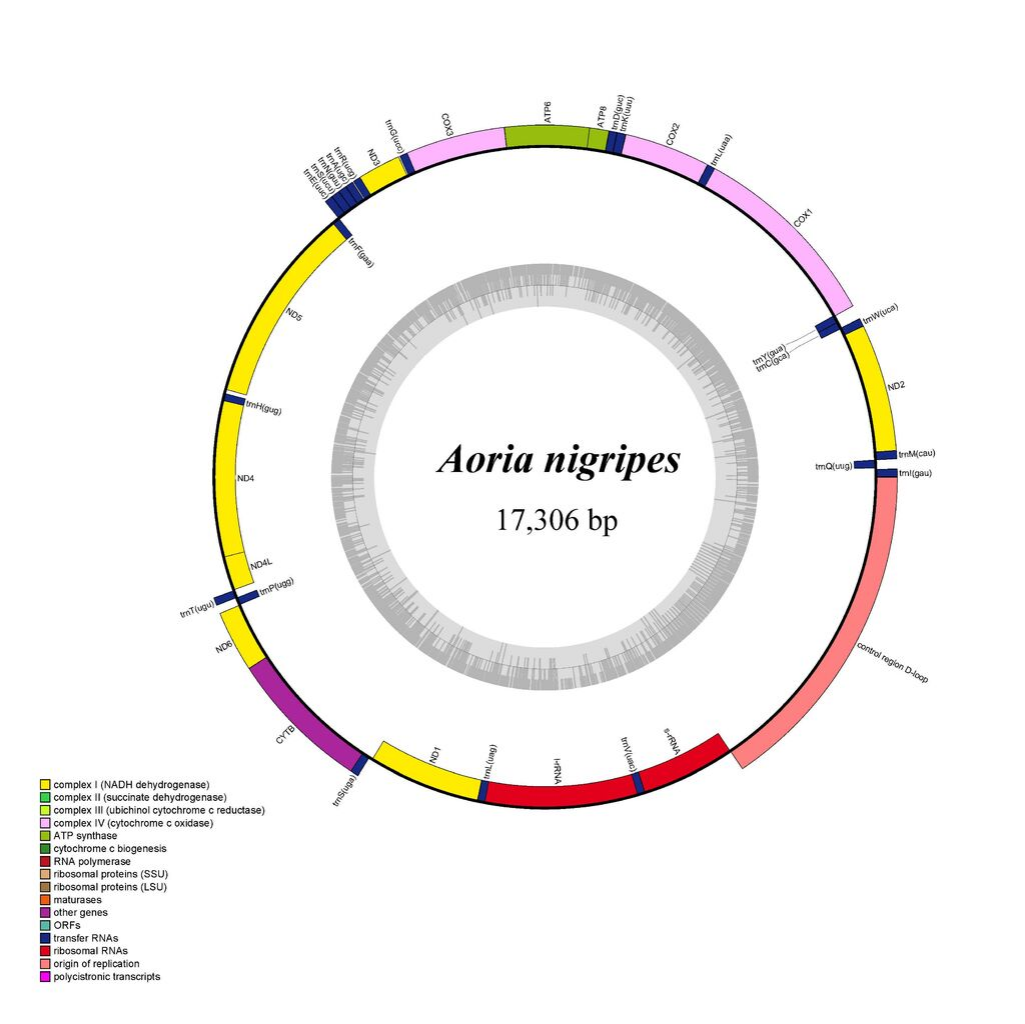
Complete mitochondrial genome map of *Aorianigripes*. The grey small circle represents the GC content graph.

The length of the complete mitogenome sequence of *A.nigripes* was 17,306 bp, which consists of 13 protein-coding genes (PCGs), 22 transfer RNA genes (tRNAs) and two ribosomal RNA genes (rRNAs) (Fig. 3). The base composition was A (41.70%), T (33.76%), G (9.01%) and C (15.53%); the A+T content was 75.46%. The 13 PCGs were 10,320 bp in total length, the longest PCG was 1530 bp (*COX1*) and the shortest was 153 bp (*ATP8*); the 22 tRNAs ranged from 62 bp to 71 bp in length; the two rRNAs were 744 bp and 1279 bp in length, respectively.

**Figure 4.**
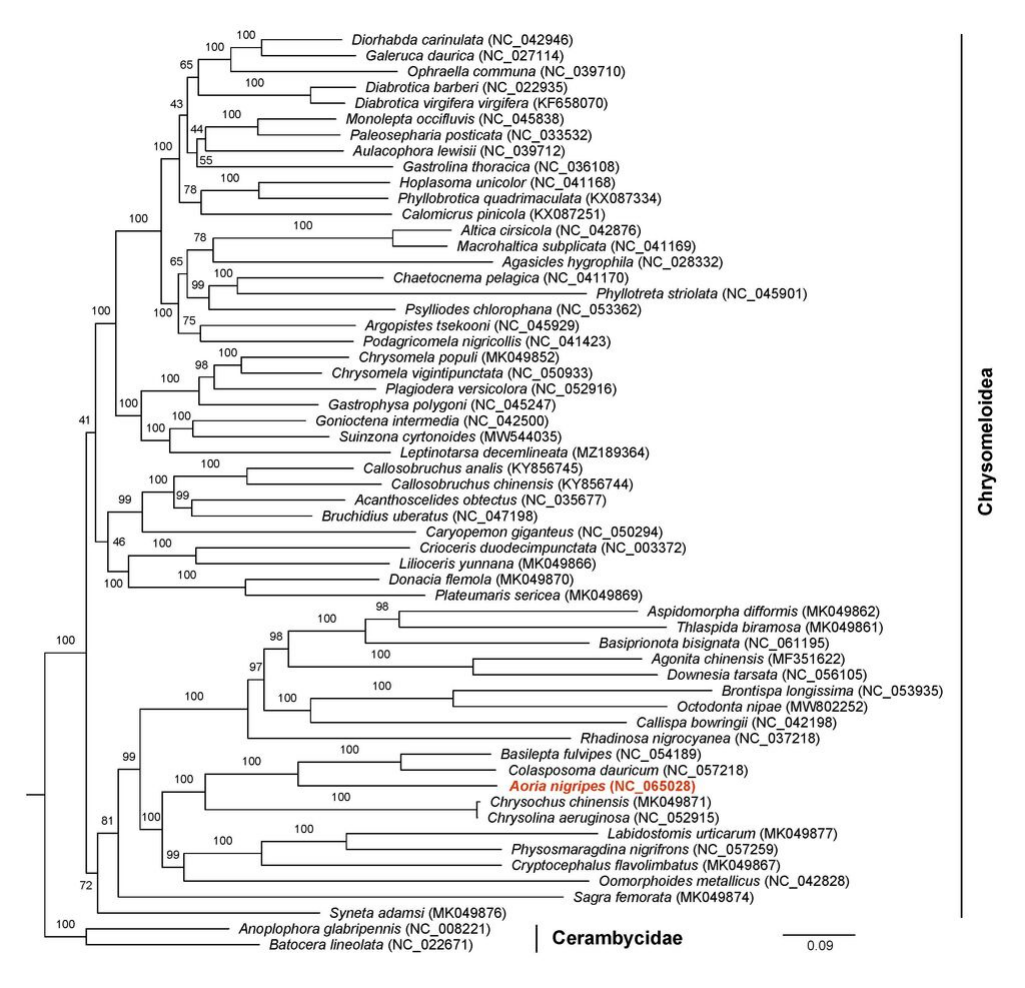
The Maximum Likelihood tree, based on the complete mitogenome sequences of 58 species. GenBank accession numbers are described in the figure. Shown next to the nodes are bootstrap support values, based on 1000 replicates.

To further understand the phylogenetic position of *A.nigripes*, we constructed the phylogenetic tree of 58 species, based on the 13 PCGs (Fig. 4); the phylogenetic analysis showed that *A.nigripes* was clustered together with *Basileptafulvipes* and *Colasposomadauricum*, which was consistent with the morphological classification status (Tan et al. 2016). In morphology, *B.fulvipes*, *C.dauricum* and *A.nigripes* showed similar characteristics with the mouth part beingof the lower mouth type. Tan et al. (2016) assigned the three species as a subfamily of Eumolpidae. The phylogenetic position of *A.nigripes* within the superfamily Chrysomeloidea was analysed, providing a molecular basis for the better study of *A.nigripes* in the future.
